# A Zebrafish Live Imaging Model Reveals Differential Responses of Microglia Toward Glioblastoma Cells *In Vivo*

**DOI:** 10.1089/zeb.2016.1339

**Published:** 2016-12-01

**Authors:** Lloyd Hamilton, Katy R. Astell, Gergana Velikova, Dirk Sieger

**Affiliations:** Centre for Neuroregeneration, University of Edinburgh, Edinburgh, United Kingdom.

**Keywords:** microglia, glioma, brain tumor, macrophages

## Abstract

Glioblastoma multiforme is the most common and deadliest form of brain cancer. Glioblastomas are infiltrated by a high number of microglia, which promote tumor growth and surrounding tissue invasion. However, it is unclear how microglia and glioma cells physically interact and if there are differences, depending on glioma cell type. Hence, we have developed a novel live imaging assay to study microglia–glioma interactions *in vivo* in the zebrafish brain. We transplanted well-established human glioblastoma cell lines, U87 and U251, into transgenic zebrafish lines with labelled macrophages/microglia. Our confocal live imaging results show distinct interactions between microglia and U87, as well as U251 glioblastoma cells that differ in number and nature. Importantly these interactions do not appear to be antitumoral as zebrafish microglia do not engulf and phagocytose the human glioblastoma cells. Finally, xenotransplants into the *irf8^−/−^* zebrafish mutant that lacks microglia, as well as pharmacological inhibition of the CSF-1 receptor (CSF-1R) on microglia, confirm a prominent role for zebrafish microglia in promoting human glioblastoma cell growth. This new model will be an important tool for drug screening and the development of future immunotherapeutics targeting microglia within glioma.

## Introduction

Gliomas are the most common type of malignant brain tumor. The most aggressive and frequently occurring form of glioma is known as glioblastoma multiforme (GBM), or high grade astrocytoma. GBM is incurable and has a median survival rate of less than 14 months.^1,2^ GBMs display extensive intra- and intertumoral heterogeneity in both morphological features and genetics.^3,4^ Furthermore, glioblastomas show a diffuse appearance, which is the main reason why these tumors cannot be cured by surgical resection. Although Scherer described the migration of glioma cells away from the main tumor mass as early as 1940,^5^ the underlying mechanisms are still not understood. Recent studies suggest that high levels of macrophages/microglia within the glioma are positively correlated with glioma grade and invasiveness.^6–17^

Microglia are the residential macrophages of the brain.^18,19^ Among many other functions, microglia sense injuries in the brain immediately, migrate to the site of the lesion, and phagocytose detrimental material.^20–23^ Strikingly, a comparable course of events is observed during glioma progression in the brain, where microglia and infiltrating macrophages are attracted to and colonize the tumor.^6,7,9,16,17^ However, instead of antitumoral activity they display protumoral functions and promote tumor growth.^11–15,24–27^ Microglia/macrophages within glioblastomas have been found to account for 30% of the glioma mass and have been implicated in establishing an immunosuppressive microenvironment.^6,7,16^ Thus, understanding the nature of microglia–glioma interactions *in vivo* could be the first step to develop future alternative strategies to interfere with glioma growth and invasiveness.

The zebrafish represents a powerful model system to explore cellular responses and molecular events *in vivo.* It has been established as a model to study various types of human cancer, ranging from B-Cell/T-Cell leukemia and melanoma to glioma.^28–38^ We have utilized a zebrafish xenotransplantation live imaging model to address microglia–glioma interactions. The zebrafish larva provides optimal characteristics that are advantageous for these studies. First, the zebrafish immune system is unique in the sense that after fertilization, the larvae survive only with the innate immune system.^39,40^ Maturation of the immune system leading to the development of the adaptive immune response occurs at between 3 and 6 weeks postfertilization.^39,40^ Thus upon xenograft these early events during tumor colonization can be studied in detail without interference by the highly diversified and complex response of an adaptive immune system. Second, a major benefit of the larval model is the optical transparency, which makes it possible to directly observe and classify the different microglia–glioma interactions *in vivo* in high resolution. To perform similar studies in a rodent model the insertion of a cranial window is necessary.^41^ While feasible, this requires an additional surgical procedure that needs to be tolerated by the animal. Furthermore, immunosuppression has to be applied upon transplantation of human cells, which might impact microglia–glioma interactions as well. To overcome this limitation, orthotopic syngeneic mouse models like the GL261 glioma model have been developed.^42^ This model, in combination with two-photon imaging, has been used very recently to monitor how microglia respond to mouse GL261 glioma cells.^43–45^ However, interactions of microglia with human glioblastoma cells have never been visualized to date.

We have exploited recently established zebrafish lines with fluorescently labelled macrophages/microglia to simultaneously monitor the migration and movement of microglia and glioblastoma cells, as well as their interactions with each other. Transplantation of human U87 and U251 glioblastoma cells into the zebrafish brain led to an immediate microglial response. To test if these responses were specific for glioblastoma cells, we performed heterotopic transplants of human fibrosarcoma cells (HT1080). Interestingly, we observed specific nonphagocytic interactions with U87 and U251 cells, which were different in number and in nature. Importantly, microglial responses toward HT1080 cells were very different and many of these cells were immediately engulfed. Finally, xenotransplants into the *irf8^−/−^* zebrafish mutant, which lacks microglia, confirmed a prominent role for microglia in promoting U87 and U251 tumor cell survival. In summary, our results show that the zebrafish larva is a powerful tool to study specific interactions between microglia and glioma cells.

## Materials and Methods

### Cell culture

Human U87MG glioblastoma cells were kindly provided by Prof Tobias Pukrop (University Hospital Regensburg, Germany). Human U251MG glioblastoma cells were purchased from CLS Cell Lines Service (Eppelheim, Germany), and human HT1080 cells were kindly provided by Dr Pamela Brown (SURF, University of Edinburgh). U87MG cells were cultured in Dulbecco's modified Eagle's medium (DMEM) containing 1% l-glutamine and supplemented with 1% (v/v) Penicillin/Streptomycin (100 mg/mL penicillin and 100 mg/mL streptomycin) and 10% (v/v) fetal calf serum at standard conditions of 100% humidity and 5% CO_2_, at 37°C. U251MG cells were cultured in DMEM containing 1% l-glutamine and supplemented with 1% (v/v) Penicillin/Streptomycin, 0.1 mM nonessential amino acids, 1 mM sodium pyruvate, and 10% (v/v) fetal calf serum at standard conditions of 100% humidity and 5% CO_2_, at 37°C. HT1080 cells were cultured in DMEM containing 30 mg/L GlutaMAX, 4.5 g/L d-Glucose, and 2.5 mM HEPES supplemented with 1% (v/v) Penicillin/Streptomycin and 10% (v/v) fetal calf serum at standard conditions of 100% humidity and 5% CO_2_, at 37°C. Cells were harvested on the day of the xenograft transplantation. The cells were washed with phosphate-buffered saline (PBS) and detached from the surface of the flask by incubation in 2 mM EDTA in PBS for 15 min at 34°C. The EDTA was removed with a 10 mL DMEM wash followed by a 10 mL PBS wash; cells were centrifuged for 1.5 min at 0.2 *g* between washes. The resulting cell pellet was resuspended in 100 μL DMEM, supplemented with phenol red (1:10 v/v), to a final concentration of 5 × 10^7^ cells/mL in preparation for the xenografts.

### Lentiviral transductions and CM-DiI dye labeling

For lentiviral transductions, cells (4.5 × 10^5^) were seeded into 60 mm dishes in a final volume of 5 mL DMEM. Cells were allowed to adhere to the dish for 4 h, at standard incubator conditions. HT1080 cells were grown on Matrigel to allow transformation. Polybrene (5 ng/mL) was added directly to the culture medium and incubated for 10 min at 37°C. The Lv-cppt-IRES-mCherry-opre viral vector (Dr. P. Brown, SURF) at a multiplicity of infection of 10 was then added to the existing media and was incubated at standard conditions for 48 h. After 48 h, the media containing viral vector were removed. The transformed cells were detached from the culture dish by 15 min of 2 mM EDTA-PBS treatment at 37°C and given a 10 mL DMEM wash and then 10 mL PBS wash; cells were centrifuged for 1.5 min at 0.2 *g* between washes. The resulting cell pellet was resuspended in 10 mL DMEM and seeded into cell culture dishes at the required density. This procedure gave rise to cell lines that stably expressed the mCherry reporter protein, which was evenly distributed throughout the cells. These mCherry expressing cells are referred to as U87, U251, and HT1080 cells throughout the text.

CM-DiI Dye (Molecular Probes) was dissolved in DMSO according to manufacturer's instructions, and a working concentration of 2 μM was used for cell labeling. Cells were incubated in 2 μM CM-DiI Dye for 10 min at 4°C. This was followed by two PBS washes and a media replacement in preparation for harvesting.

### Mammalian cell growth kinetics

The growth rates of HT1080 mCherry, U87 mCherry, and U251 mCherry cells at 34°C compared to 37°C were determined over a 4-day period. Monolayer cultures of each cell line were harvested, using Accutase (Sigma-Aldrich), when ∼75% confluent. The detached cells were washed once with 1 × PBS and resuspended in culture medium. The cells were counted using a Neubauer counting chamber (depth 0.1 mm, 1/400 mm^2^; Hawksley), and 1500 cells, in 1 mL, of culture medium were transferred into the wells of 12-well plates. Cells were cultured continuously for up to 4 days, without replenishing media, at either 34°C or 37°C under normoxic conditions. Each day (at 24-h intervals), the cell number, in three individual wells, was counted for each cell line and the average cell number was calculated. The experiment was repeated in triplicate, and the average cell numbers, from the three experiments, were plotted to produce growth curves.

### Zebrafish maintenance, xenografts, and pharmacological treatments

**FIG. 1.**
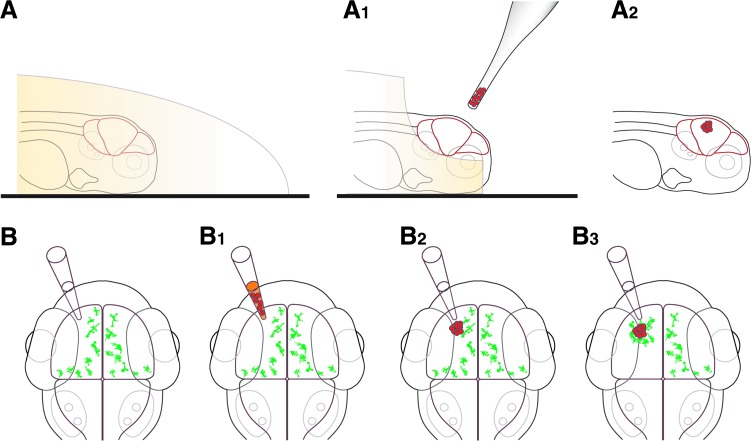
Zebrafish larval xenograft. **(A–A_2_)** Schematic representation of the xenograft technique, lateral view. **(A)** Zebrafish larvae were mounted in low-melting point agarose (beige). **(A_1_)** Larval heads were released from the low-melting point agarose to allow cell transplantation into the brain. **(A_2_)** Cells (*red*) were transplanted into the optic tectum. **(B–B_3_)** Schematic representation of the xenograft technique, dorsal view. Cells (*red*) were transplanted into the left optic tectum, stimulating a microglia (*green*) response (depending on cell type). The right optic tectum served as an internal control.

Zebrafish were housed in a purpose built zebrafish facility, in the Queen's Medical Research Institute, maintained by the University of Edinburgh Biological Resources. All zebrafish larvae were kept at 28°C on a 14-h light/10-h dark photoperiod. Embryos were obtained by natural spawning from adult mpeg1:EGFP^46^ and *irf8^−/−^*^47^ zebrafish strains. Animal experimentation was approved by the Ethics Review Committee of the University of Edinburgh and the Home Office, in accordance with the Scientific Procedure Act 1986. Embryos were treated with 200 μM 1-phenyl 2-thiourea (PTU) from the end of the first day of development for the duration of the experiment to inhibit pigmentation. Xenografts were conducted on larvae that had been dechorionated and anesthetized with 2.5 mM Ethyl 3-aminobenzoate methanesulfonate (Tricaine) in zebrafish embryonic medium (6.4 mM KCl, 0.22 mM NaCl, 0.33 mM CaCl_2_ 2H_2_O, 0.33 mM MgSO_4_ 7H_2_O), at 3 dpf. Anesthetized zebrafish larvae were immobilized in 1.5% (w/v) low-melting point agarose solution made with zebrafish embryonic medium. The heads of the embryos were carefully exposed from the agarose before transplantation (Fig. 1). All transplantations were performed using a FemtoJet 4i microinjector (Eppendorf, Hamburg, Germany). Transplantation needles were prepared from borosilicate glass capillaries, lacking an internal filament, using a glass micropipette puller (Sutter Instrument Co. P-87, Novato). U87/U251/HT1080 cells (*n* = 8–30) were transplanted into the optic tectum of each zebrafish larva. Individual zebrafish larvae containing U87/U251/HT1080 cells were maintained in 12-well plates, containing 4 mL zebrafish embryo medium supplemented with 200 μM PTU, at 34°C. For pharmacological treatments, U87 cell transplanted larvae were maintained in 24-well plates in zebrafish embryo medium supplemented with 200 μM PTU, at 34°C. Transplanted larvae were treated from 4 dpf (1 day posttransplantation) with either 1% DMSO (control) or 1% DMSO/25 μM BLZ945 for 5 days. Larvae were imaged at 4 dpf before the start of treatment to obtain microglial cell counts and U87 cell counts. Treated larvae were kept individually and imaged at the end of treatment (9 dpf) to obtain final microglia and U87 cell counts.

### Imaging

Zebrafish larvae were anesthetized with 2.5 mM tricaine in embryonic medium and mounted in 1.5% (w/v) low-melting point agarose. Time lapse and still images were obtained using an Andor spinning disk confocal fluorescent microscope with a 20 × /NA 0.75 Olympus objective lens. The acquired z stacks for both single time point and time lapse images were selected to encompass all regions of interest within the zebrafish larval brain. Time lapse imaging was conducted for a period of 14–17 h with the time interval between images set at 3 min. Single time point images were obtained at 0–4 days posttransplantation (dpt).

### Analysis

Analysis of all images was conducted using Imaris (Bitplane, Belfast, United Kingdom). All data were plotted using GraphPad and analyzed using one way ANOVA and *post hoc* analysis with Sidak's multiple comparisons test. The cell number was determined for each image by manual counting in 3D. These values were used to plot survival curves for each cell type in the different zebrafish lines. The survival curve was determined as the ratio expressed as a percentage between the cell number at each time point and the starting cell number.

The number of microglia interactions between individual microglia and the transplanted cells was measured by counting the number of direct interactions between both cell types and normalizing for the number of xenografted cells at the respective time point. Interactions were defined as direct contacts between microglial surfaces and transplanted cell surfaces, including engulfment and phagocytosis of transplanted cells (Supplementary Fig. S1B–E; Supplementary Data are available online at www.liebertpub.com/zeb). If a microglial cell showed several protrusions in contact with a single transplanted cell, this was counted as one interaction between these two cells.

The invasiveness of the different cell types was determined by measuring the spread of fluorescently labelled cells in 3D within the zebrafish larval brain over time. Using Imaris, a 3D sphere was created to encompass all fluorescent cells of interest. Transplanted cells that had been engulfed by macrophages/microglia were not included within the sphere (see Results section; Supplementary Fig. S2C′, D′). The diameter of the created 3D sphere was used to measure invasiveness. Invasiveness was determined as a percentage between the final diameter of the sphere, required to encompass the transplanted cells 4 dpt compared to the diameter of the sphere surrounding the initial cell mass directly after transplantation.

## Results

### *In vivo* imaging reveals different growth patterns and microglial responses for U87 and U251 glioblastoma cells in the larval zebrafish brain

The combination of fluorescent transgenic tools and the innate optical transparency of the zebrafish larva makes it possible to study microglial interactions under high temporal and optical resolution in the living brain.^23,48–50^ To study microglial responses to xenografted human glioblastoma cells, we made use of the mpeg1:EGFP transgenic zebrafish, in which all macrophages, including microglia, can be visualized and tracked.^46^ The xenotransplantation protocol used in this study is an optimized version of our previously developed injection protocol and is minimally invasive.^23^ Briefly, larvae were mounted in low-melting point agarose, and their heads were carefully exposed from the agarose using a sharp needle before transplantation (Fig. 1A–A_2_). Transplantations were performed into the left optic tectum using a glass capillary (Fig. 1B–B_3_). Sham injections of culture media led only to a short injury response of microglial cells that were in direct proximity to the injection site. This injury response was usually resolved within a few hours (data not shown). To study microglial responses to glioblastoma cells, we transplanted human-derived U87mCherry (referred to as U87) and U251mCherry (referred to as U251) glioblastoma cells into the optic tectum and whole brain confocal imaging was performed at 0–4 dpt. To test if microglial responses were specific for glioblastoma cells, we performed heterotopic transplants of the human fibrosarcoma cell line HT1080mCherry (referred to as HT1080) and performed imaging at the same time points.

**FIG. 2.**
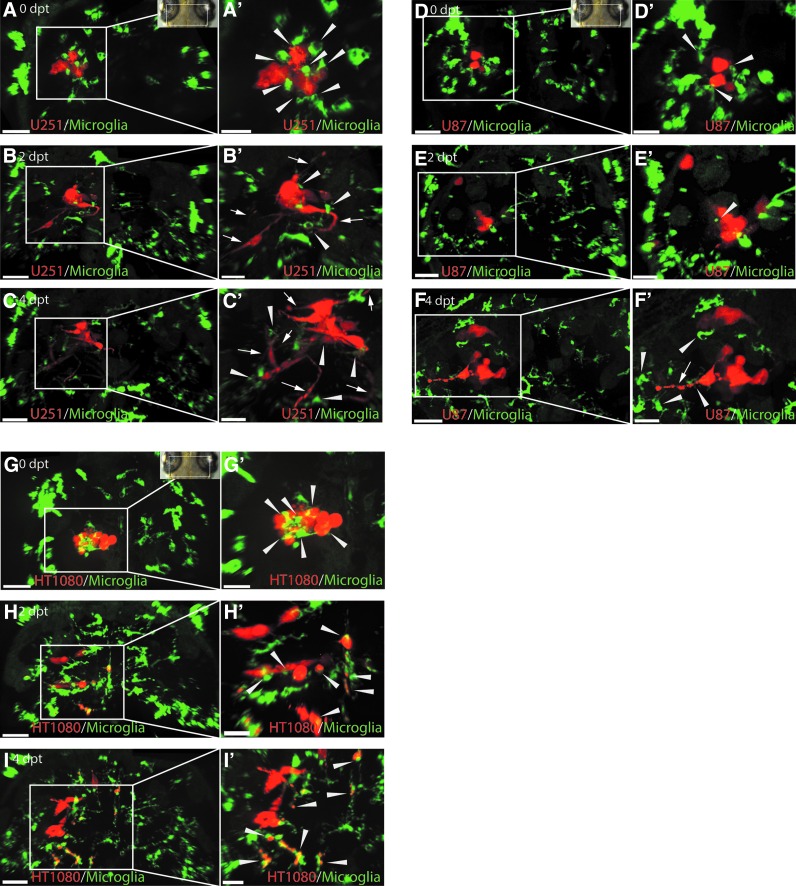
U251, U87, and HT1080 cells exhibit different growth patterns in the zebrafish optic tectum and stimulate an intensive microglia response. Representative confocal images of the optic tectum of mpeg1:EGFP zebrafish transplanted with U251mCherry (U251) glioblastoma cells, U87mCherry (U87) glioblastoma cells, and HT1080mCherry (HT1080) fibrosarcoma cells at 3 dpf. **(A–C)** Images from *top* to *bottom* are in chronological order showing 0, 2, and 4 dpt of U251 cells; **(A**′**–C**′**)** Higher magnification of regions of interest in **(A–C)** to highlight interactions of microglia (*green*) and U251 cells (*red*). U251 cellular projections are marked with a *white arrow*. Microglia interacting with U251 cells are marked with a *white arrowhead*. **(D–F)** Images from *top* to *bottom* are in chronological order showing 0, 2, and 4 dpt of U87 cells; **(D**′**–F**′**)** Higher magnification of regions of interest in **(D–F)** to highlight interactions of microglia (*green*) and U87 cells (*red*). U87 cellular projections are marked with a *white arrow*. Microglia interacting with U87 cells are marked with a *white arrowhead*. **(G–I)** Images from *top* to *bottom* are in chronological order showing 0, 2, and 4 dpt of HT1080 cells; **(G**′**–I**′**)** Higher magnification of regions of interest **(G–I)** to highlight interactions of microglia (*green*) and HT1080 cells (*red*). Microglia interacting with HT1080 cells are marked with a *white arrowhead*. Scale bars for **(A–I)**: 50 μm. Scale bars for **(A**′**–I**′**)**: 30 μm. All images represent maximum intensity projections of confocal stacks. Images were captured using an Andor spinning disk confocal microscope with a 20 × /NA 0.75 objective. *Insets* in **(A, G, and D)** show the orientation of samples (anterior to the *top*) and the region imaged (*white rectangle*). dpf, days postfertilization; dpt, days posttransplantation

Upon transplantation of U87 and U251 glioblastoma cells into the larval zebrafish tectum, the cells invaded the surrounding tissue of the optic tectum moving away from the initial transplantation site (Fig. 2). However, it was immediately obvious that U251 cells and U87 cells had very different cellular morphologies within the zebrafish brain (Fig. 2). U251 glioblastoma cells showed infiltrative cellular protrusions that projected from the main cell bodies into the surrounding tissue (Fig. 2B′, C′, arrows). In most U251 samples, it was observed that these projections extended to over 100 μm in length and formed interactions with microglia outside of the central glioma mass (Fig. 2C′, arrows, arrowheads). Furthermore, U251 cells attracted many microglia that stayed within the vicinity of the U251 cells over the duration of the experiment (Fig. 2A–C). These microglia showed different types of interactions with U251 cells (see below) that could be observed over the full duration of the experiment (Fig. 2A′–C′, arrowheads).

Transplanted U87 cells showed a different morphology compared to U251 cells (Fig. 2D–F). The borders of the main U87 glioblastoma cell mass were more defined, and cellular protrusions were observed to a much lesser degree compared to the U251 cells (compare Fig. 2C′, arrows to Fig. 2F′, arrows). Nevertheless, single-invasive U87 cells were observed outside the main cell mass (Fig. 2E′, F′). U87 cells also attracted microglia and interactions between the U87 cells and microglia (see below) were observed at all time points (Fig. 2D′–F′, white arrow heads).

Interestingly, heterotopic transplants of human HT1080 cells into the larval zebrafish brain resulted in a different outcome. HT1080 cells attracted many microglia (Fig. 2G′) and these microglia immediately engulfed HT1080 cells (Fig. 2H′, I′ and Supplementary Fig. S1B). Thus after 4 dpt, many HT1080 cells were detected within phagosomes of microglia, while less than half of the HT1080 cells escaped engulfment by microglia (Fig. 2I′ and Supplementary Fig. S1B).

### Microglia differently interact with U87 and U251 cells

**FIG. 3.**
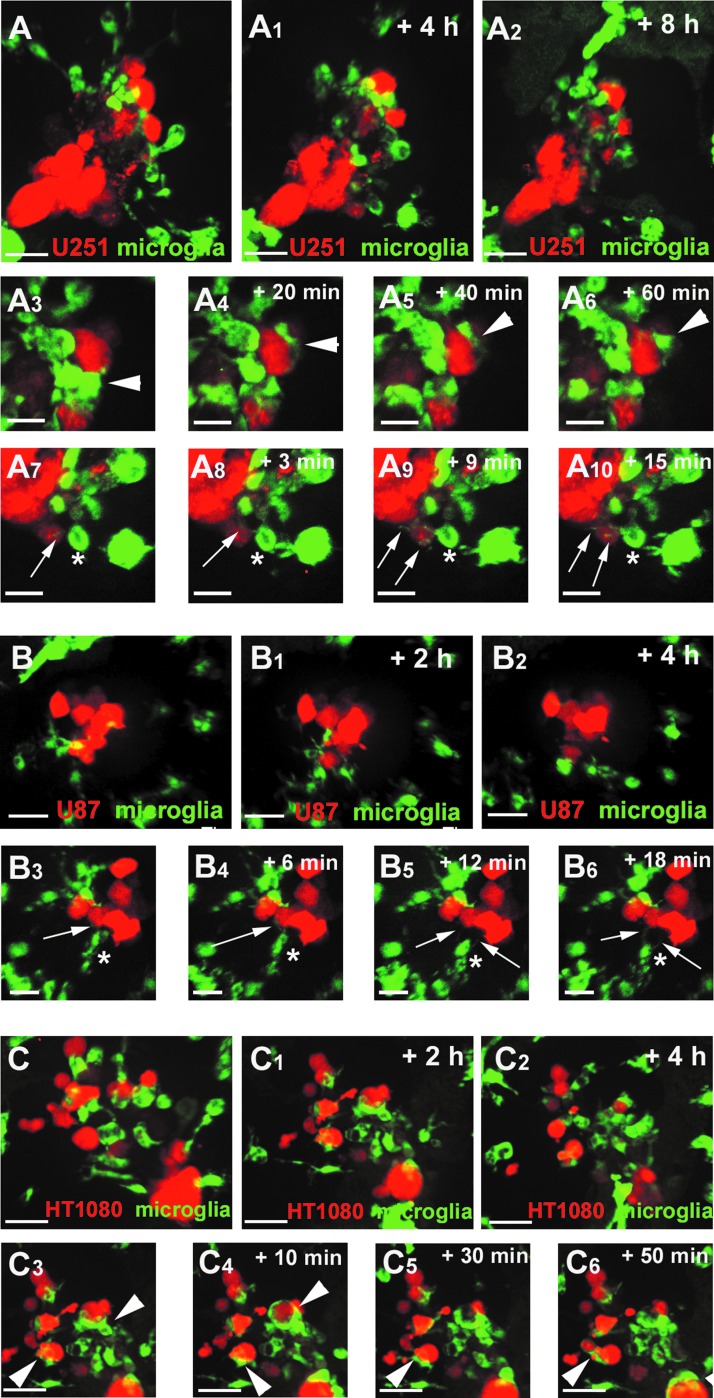
Microglia interact differently with U251, U87, and HT1080 cells. **(A)** Confocal time series showing microglia (*green*) responding to U251 cells (*red*) in the zebrafish optic tectum (recording times indicated; see also Supplementary Movie S1). **(A_3_–A_6_)** Time series highlighting a microglial cell closely interacting with a U251 cell for more than 60 min. *White arrowhead* marks the microglial cell interacting with the U251 cell. **(A_7_–A_10_)** Time series highlighting a microglial cell constantly sending and retracting processes toward U251 cells. *White asterisk* marks a microglial cell sending and retracting processes. *White arrows* mark microglial processes toward U251 cells. Scale bars for **(A–A_2_)**: 30 μm. Scale bars for **(A_3_–A_10_)**: 15 μm. **(B)** Confocal time series showing microglia (*green*) responding to U87 cells (*red*) in the zebrafish optic tectum (recording times indicated; see also Supplementary Movie S2). **(B_3_–B_6_)** Time series highlighting a microglial cell closely interacting with U87 cells. *White asterisk* marks a microglial cell sending and retracting processes. *White arrows* mark microglial processes toward U87 cells. Scale bars for **(B–B_2_)**: 30 μm. Scale bars for **(B_3_–B_6_)**: 20 μm. **(C)** Confocal time series showing microglia (*green*) responding to HT1080 cells (*red*) in the zebrafish optic tectum (recording times indicated; see also Supplementary Movie S3). **(C_3_–C_6_)** Time series highlighting microglia engulfing and phagocytosing HT1080 cells. *Arrowheads* indicate microglia engulfing and phagocytosing HT1080 cells. Scale bars for **(C–C_6_)**: 30 μm. All images represent maximum intensity projections of confocal stacks. Images were captured using an Andor spinning disk confocal microscope with a 20 × /NA 0.75 objective.

To analyze the nature of interactions between microglia and the different transplanted cell types in more detail, we performed live imaging of U87, U251, and HT1080 transplanted mpeg1:EGFP fish at 3-min intervals for up to 14 h. These time series revealed obvious differences in the responses of microglia toward the different cell types. Interestingly, microglia behavior was very different upon transplantation of U87 and U251 cells. U251 cells immediately attracted many microglia (Fig. 3A and Supplementary Movie S1). These microglia deeply infiltrated the U251 cell mass and exhibited continuous interactions over the remaining hours (Fig. 3A and Supplementary Movie S1). We observed two main types of cellular surface interactions. The first type of interaction observed was a long-lasting interaction between individual microglia and single U251 cells (Fig. 3A_3_–A_6_ [arrowhead] and Supplementary Movie S1). Here microglia flatten their surfaces against the surface of the U251 cells and keep this close position for hours while slightly moving on the surface of the U251 cells (Fig. 3A_3_–A_6_ [arrowhead] and Supplementary Movie S1). The second type of interaction observed was of dynamic nature, reflected in constant extensions and retractions of microglial processes that made contact with the surface of U251 cells (Fig. 3A_7_–A_10_ [asterisk, arrows] and Supplementary Movie S1). These contacts were of short duration (minutes) and individual microglia interacted with several U251 cells. In comparison to U251 cells, U87 cells attracted a lower number of microglial cells and they did not penetrate the U87 cellular mass (Fig. 3B and Supplementary Movie S2). Despite this difference, we detected interactions between the microglia and transplanted U87 cells. Microglia remained at the margin of the U87 cell mass and sent out and retracted cellular protrusions to make contact with the U87 cells (Fig. 3B_3_–B_6_ [asterisks, arrows]). As observed for U251 cells, these contacts were of short duration and individual microglia interacted with several U87 cells (Supplementary Movie S2).

**FIG. 4.**
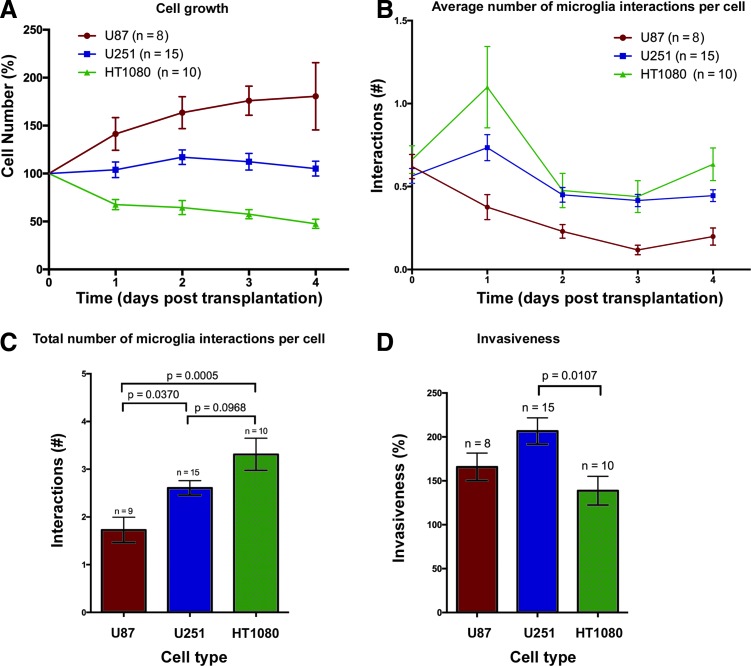
Growth and invasiveness of U87, U251, and HT1080 cells are accompanied by a different number of microglial interactions. U87, U251, and HT1080 cells were transplanted into the larval zebrafish tectum at 3 dpf, and larvae were imaged at 0–4 dpt. 3D images were analyzed using Imaris (see Materials and Methods section). **(A)** Survival of U87, U251, and HT1080 cells over 4 days displayed as the ratio (expressed as a percentage) between the cell number at each time point and the starting cell number. *p* Values for the different time points are: 1 dpt U87 versus U251, *p* = 0.0454; U87 versus HT1080, *p* = 0.0002; U251 versus HT1080, *p* = 0.0366; 2 dpt U87 versus U251, *p* = 0.0044; U87 versus HT1080, *p* < 0.0001; U251 versus HT1080, *p* = 0.1352; 3 dpt U87 versus U251, *p* = 0.0003; U87 versus HT1080, *p* < 0.0001; U251 versus HT1080, *p* = 0.0008; 4 dpt U87 versus U251, *p* = 0.0111; U87 versus HT1080, *p* = 0.0419; and U251 versus HT1080, *p* = 0.0419. **(B)** Average number of microglial interactions per transplanted cell from day 0 to day 4 posttransplantation for U87, U251, and HT1080 cells. **(C)** Mean of the total number of microglial interactions for U87, U251, and HT1080 cells per transplanted cell in mpeg1:EGFP zebrafish over 4 days. **(D)** Mean of cell invasiveness in percent. *n* numbers represent the number of larvae. All error bars represent the standard error of the mean. *p* values are indicated on graph.

As stated before, the microglial response toward HT1080 cells was very different. Following transplantation of HT1080 cells into the larval zebrafish brain, the main microglial response resulted in the immediate engulfment of many of these cells (Fig. 3C and Supplementary Movie S3). Over the time course, several microglia cells were found to contain HT1080 cellular fragments within their phagosomes (Fig. 3C_3_–C_6_ [arrowheads] and Supplementary Fig. S1B–SB_4_). This explains the decrease in HT1080 cell numbers that we observed over the 4-day time course (Fig. 4A).

### U87 and U251 glioma cells show differences in growth and number of microglia interactions in the larval zebrafish brain

To investigate the growth of the different cell types upon transplantation, we performed cell counts based on the confocal z-stacks acquired at the different time points. These results show that U87 survival was significantly higher at all time points compared to both U251 and HT1080 (Fig. 4A). Over the course of the experiment, U87 cell numbers increased to 180% ± 35.1%, while U251 cell numbers remained constant (Fig. 4A). The observed difference in growth for U87 cells and U251 cells upon transplantation is in line with their *in vitro* growth rates. The comparison of growth rates for these cells at 37°C and 34°C *in vitro* showed that U87 cells grow much faster than U251 cells at both 37°C and 34°C (Supplementary Fig. S1A). In comparison, HT1080 cell numbers decreased to 47.6% ± 4.8% at 4 dpt in the zebrafish larval tectum (Fig. 4A). This is in contrast to their growth *in vitro* as HT1080 cells were observed to grow faster than U251 cells at 37°C and 34°C (Supplementary Fig. S1A). Thus, orthotopic transplants of U87 and U251 glioblastoma cells into the optic tectum of the zebrafish brain show a greater rate of survival compared to heterotopic HT1080 transplants.

Although glioma infiltration by microglia has been described in many studies, interactions between microglial cells and the glioma cells have only been described recently *in vivo* for a mouse GL261 glioma model.^43–45^ Microglial interactions with human glioblastoma cells have never been directly visualized *in vivo* at high resolution until now. To ascertain if the different transplanted human glioma cell types stimulate a different number of microglia interactions, the number of individual microglia cells in direct contact with transplanted cells was analyzed based on the confocal stacks acquired between 0 and 4 dpt (see Materials and Methods section and Fig. S1C, E).

U251 glioblastoma cells stimulated a significantly higher number of microglia interactions compared to U87 (Fig. 4B, C). One way ANOVA of the total number of microglial interactions over the whole 4-day period indicated a significant difference in the number of microglial interactions with U251 cells (2.61 ± 0.150, *n* = 15) compared to U87 cells (1.73 ± 0.264, *p* = 0.037, *n* = 8) (Fig. 4C). HT1080 cells stimulated a high number of microglial interactions (3.31 ± 0.338, *n* = 10; Fig. 4B, C) compared to U87 (1.73 ± 0.264, *p* = 0.0005, *n* = 8). However, microglia-HT1080 cell interactions were very different in nature compared to interactions with U87 and U251 cells and included a high degree of engulfment of HT1080 cells.

We have shown that transplanted U87, U251, and HT1080 cells differ in survival rates and microglial responses. To test if these cell types show differences in their infiltrative behavior, we measured their invasiveness in 3D over time. This was achieved by creating a 3D sphere that accurately encompassed the transplanted cells at the different time points (see Materials and Methods section and Supplementary Fig. S2D). The diameter of the created 3D sphere was used to measure invasiveness. By calculating the fold increase between spheres on sequential days, a valuation of invasiveness could be obtained. Based on this method, we compared the invasiveness of the different cell types. Interestingly, although U87 cells showed the best rate of survival in our experiments (Fig. 4A), U251 cells were the most invasive cells. U251 cells showed the highest rate of surrounding tissue infiltration (206.7% ± 11.2%, *n* = 15) compared to HT1080 cells (138.8% ± 16.5%, *p* = 0.0107, *n* = 10) and U87 cells (165.9% ± 15.7%, *p* = 0.203, *n* = 8) (Fig. 4D). Importantly, the results observed in this study coincide with previous histopathological studies conducted in rodent models, which documented the differences in invasiveness between the two glioblastoma cell lines.^51–53^

Of note in this study, and of importance to measure invasiveness accurately, these measurements have to be done in 3D, as 2D projections of confocal stacks do not reflect the total invasiveness in all dimensions. Furthermore, cells need to be stably expressing the fluorophore as trials using dye labelled cells (CM-DiI Dye) showed false positive rates of invasiveness due to detection of small fluorescent particles that may be either pure dye aggregates or vesicles released by tumor cells (Supplementary Fig. S2A, B). These small dye aggregates spread within the tissue in all dimensions, thus, including these particles leads to misleading results and an overestimation of invasiveness. Furthermore, an important prerequisite to measure invasiveness precisely is an additional labeling for microglia. Depending on the cell type, transplanted cells might be engulfed by microglia and signals will be detected within microglial phagosomes. These signals do not represent viable cells, and thus, do not reflect true infiltration. Only the additional microglial labeling allows the identification of these engulfed cells to exclude them from the analysis (Supplementary Fig. S2C, D).

### Microglia promote U87 and U251 cell growth

To further investigate the direct role of microglia–glioma interactions in promoting survival, proliferation, and invasiveness of glioma cells, xenografting experiments were conducted in *irf8^−/−^* zebrafish that lack macrophages/microglia. Interferon regulatory factor 8 (*irf8*) is vital for macrophage development in mammals and in teleosts.^47^ The *irf8* null mutant (*irf8^−/−^*) zebrafish was characterized to lack macrophages up to around 7 dpf and microglia were absent in the brain until 31 dpf.^47^ Thus we investigated xenografted U87, U251, and HT1080 cell survival and invasiveness in *irf8^−/−^* mutant zebrafish and compared the results to those observed in our mpeg1:EGFP xenograft studies.

**FIG. 5.**
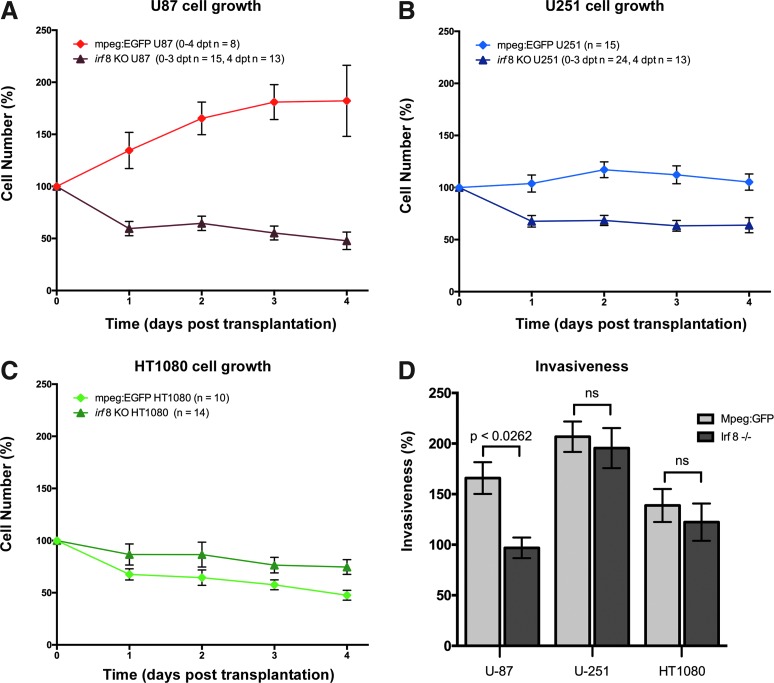
Zebrafish microglia promote survival of U87 and U251 cells. U87, U251, and HT1080 cells were transplanted into the larval zebrafish tectum of mpeg1:EGFP and *irf8^−/−^* zebrafish at 3 dpf and larvae were imaged at 0–4 dpt. **(A)** Comparison of survival for U87 cells in mpeg1:EGFP and *irf8^−/−^* zebrafish over 4 days displayed as the ratio (expressed as a percentage) between the cell number at each time point and the starting cell number. **(B)** Comparison of survival for U251 cells in mpeg1:EGFP and *irf8^−/−^* zebrafish over 4 days displayed in as the ratio (expressed as a percentage) between the cell number at each time point and the starting cell number. **(C)** Comparison of survival for HT1080 cells in mpeg1:EGFP and *irf8^−/−^* zebrafish over 4 days displayed as the ratio (expressed as a percentage) between the cell number at each time point and the starting cell number. **(D)** Comparison of the mean of cell invasiveness in mpeg1:EGFP and *irf8^−/−^* zebrafish. *n* numbers represent the number of fish. All error bars represent the standard error of the mean. *p* values are indicated on graph (ns = nonsignificant).

Xenografts in *irf8^−/−^* zebrafish highlighted a strong impact of microglia on tumor progression for U87 cells and U251 cells. The results showed a significant reduction of U87 cell survival at all time points in *irf8^−/−^* mutants compared to mpeg1:EGFP (Fig. 5A). At 4 dpt the survival of U87 cells was 132% ± 16% (*p* < 0.0001) lower in *irf8^−/−^* mutants compared to mpeg1:EGFP (Fig. 5A). In line with this, we detected a significant decrease in tissue infiltration for U87 cells in *irf8^−/−^* zebrafish (96.9% ± 10.2%) versus mpeg1:EGFP (165.9% ± 15.7%) (Fig. 5D). The survival of U251 cells in *irf8^−/−^* zebrafish larvae was also significantly reduced (Fig. 5B). At 4 dpt the survival of U251 cells was 41.4% ± 11.62% (*p* = 0.002) lower in *irf8^−/−^* mutants compared to mpeg1:EGFP (Fig. 5B). However, U251 tissue infiltration was not affected in *irf8^−/−^* zebrafish (195.5% ± 19.8%) compared to mpeg1:EGFP (206.7% ± 15.0%) (Fig. 5D).

Transplantation of HT1080 cells into *irf8^−/−^* zebrafish showed contrasting results. While HT1080 survival was rather poor in mpeg1:EGFP fish, a trend was detected toward better survival in the microglia mutant background (Fig. 5C). The results showed a nonsignificant increase of HT1080 cell survival by 27.1% ± 12.1% (*p* = 0.333) at 4 dpt in *irf8^−/−^* mutants compared to mpeg1:EGFP (Fig. 5C). Although better survival of HT1080 cells was observed in *irf8^−/−^* zebrafish, we did not detect an increase in tissue infiltration for HT1080 in the microglia mutant background (Fig. 5D).

**FIG. 6.**
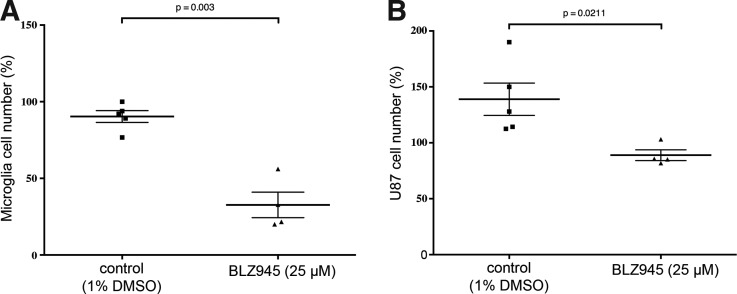
Inhibition of CSF-1R signaling leads to reduced microglia numbers and impairs U87 cell growth. U87 cells were transplanted into the larval zebrafish tectum of mpeg1:EGFP zebrafish at 3 dpf. Transplanted larvae were treated with 1% DMSO or 1% DMSO/25 μM BLZ945 from 4 dpf for 5 days. Microglial cell counts and U87 cell counts were done at 4 dpf before the start of treatment and the end of treatment (9 dpf). **(A)** Comparison of microglial cell numbers in control (1% DMSO) and BLZ945-treated zebrafish. Microglia numbers are displayed as the ratio (expressed as a percentage) of the final numbers at 9 dpf divided by the starting number at 4 dpf. **(B)** Comparison of U87 cell numbers in control (1% DMSO) and BLZ945-treated zebrafish. U87 cell numbers are displayed as the ratio (expressed as a percentage) of the final numbers at 9 dpf divided by the starting number at 4 dpf. All error bars represent the standard error of the mean. *p* values are indicated on graph.

To test if the protumoral function of the microglia can also be altered by pharmacological means in our xenograft model, we used the CSF-1R inhibitor BLZ945. CSF-1R signaling is crucial for survival of macrophages and microglia. BLZ945 has recently been shown to target microglia in a mouse proneural GBM model, leading to a regression of established tumors.^26^ To test BLZ945 in our model, we transplanted mpeg1:EGFP larvae with U87 cells at 3 dpf and incubated these larvae from 4 dpf (1 dpt) in 25 μM BLZ945 for 5 days. These larvae developed normally and did not show any adverse effects, confirming the high specificity for BLZ945 for the CSF-1R. Indeed, treatment with BLZ945 led to reduction of total microglial numbers in the optic tectum and to a decrease in U87 cell growth compared to controls (Fig. 6). Interestingly, as BLZ945 treatment did not lead to an ablation of all microglia, the impact on U87 cell growth was not as strong as observed before in the *irf8^−/−^* zebrafish (compare Figs. 5A and 6).

Together, these results clearly show that zebrafish microglia support the growth of human glioblastoma cells and, thus, further highlight the suitability of the zebrafish model to study human glioblastomas.

## Discussion

### The zebrafish as a model to study microglial responses toward glioma cells

The xenografting live-imaging assay used in this study presents a novel system that allows the response of the entire microglial network during glioma colonization to be studied in the living brain. Previous research has already demonstrated the power of the zebrafish model to study glioma growth. Several studies have transplanted glioma cells into the zebrafish yolk and shown that these cells form tumors, induce angiogenesis, and are responsive to treatment.^54–56^ Furthermore, intracranial models of glioma in larval zebrafish have been developed to study glioma invasiveness and angiogenesis^37^ and to use the zebrafish as a powerful model for drug screening.^38,57^ However, none of these studies have investigated the microglial response to xenografted cells. Thus we have established a larval zebrafish model to study microglia–glioma interactions in real time in the living brain.

We show that transplantation of U87, U251, and HT1080 cells into the optic tectum of larval zebrafish stimulated a microglia response that resulted in the recruitment of microglia cell bodies to the site of transplantation. The injection itself is minimally invasive, and media only with sham injections show a short injury response by microglial cells in direct proximity to the injection site. This microglial response is usually resolved within a few hours. However, the interactions observed upon xenotransplant lasted throughout the 4 days of imaging and were intimate. We observed that microglial cell bodies, residing outside of the transplantation site, extended projections that interacted with the glioma and fibrosarcoma cell surfaces. Furthermore, our assay clearly stresses the importance of orthotopic transplants, as microglial responses toward heterotopic transplants were very different. Importantly, microglia were observed to engulf many of the HT1080 cells immediately, which is in line with the low survival rates we observed for HT1080 cells. This indicates that HT1080 cells trigger different signaling cascades leading to an increase in microglial phagocytosis. Indeed, HT1080 cells show a very different gene expression profile compared to U87 and U251 cells (Genevestigator^58^ differential gene expression comparison, not shown). Among the many differently regulated genes, several genes coding for cytokines and cell surface proteins can be found, including the lymphotoxin β receptor (LTβR), for example. LTβR shows higher expression levels in HT1080 cells compared to U87 and U251 cells. The activation of LTβR has been shown to induce IL-8 expression and release, leading to a pro-inflammatory environment and the attraction of phagocytes.^59^ Furthermore, HT1080 cells show higher expression levels of IL-1α compared to U87 and U251 cells. IL-1α has been reported to induce expression of IL-6, IL-8, monocyte chemotactic protein-1, and granulocyte macrophage colony-stimulating factor expression and, thus, to trigger inflammation.^60^ These signals and others might lead to a pro-inflammatory activation of the microglia and to an increase in their phagocytic activity.

Interestingly, for U87 and U251 glioma cells, we observed distinct interactions with the microglia. U251 cells stimulated a higher number of microglial interactions compared to U87 cells. Microglia infiltrated the U251 cell mass and intimately interacted with U251 cells over the full duration of the experiment. Furthermore, in U251 samples, microglia were often seen to interact with U251 protrusions in the deeper tissue outside of the glioma central mass. In comparison, U87 cells attracted fewer microglia and these microglia showed only superficial interactions with U87 cells that diminished over time. These interactions were presented by individual microglial cells, which extended and retracted their protrusions to make surface contacts with the U87 cells. The differences in microglial responses toward U87 cells and U251 cells highlight intrinsic differences between these cell types. U251 cells are mutant for the tumor suppressor p53 and show high levels of expression for GFAP, vimentin, and S100β, for example.^51^ In contrast, U87 cells express wild-type tumor suppressor p53 and lack GFAP or S100β expression.^51^ Furthermore, U251 cells have been shown to express high levels of CCL2, which stimulates microglial responses.^14^ The low levels of CCL2 in U87 cells might be one of the reasons for the lower number of interactions with microglia cells observed in this study.

Our new model will facilitate new studies to reveal if microglia respond differently depending on glioblastoma subtype. Based on their gene expression, glioblastomas are classified into the Proneural, Neural, Mesenchymal, or Classical subtype.^61^ Interestingly, differential expression levels of immune genes have been detected between these glioblastoma subtypes.^62^ Thus, it is tempting to speculate that microglia recruitment and activation will vary depending on the molecular glioblastoma subtype.

### Zebrafish microglia promote survival and invasiveness of human glioblastoma cells

U87 and U251 cells showed differences in growth and invasiveness upon transplantation into the zebrafish tectum. These differences are in line with differences in their *in vitro* growth rates and differences in invasiveness upon transplantation in rodent models.^53^ When investigating how zebrafish microglia activity affected these attributes, U87 and U251 glioblastoma cells were clearly influenced by microglia. This was demonstrated by the significant decrease in survival and invasiveness of U87 cells in the *irf8^−/−^* zebrafish. In addition, U251 cell survival was also significantly decreased in the *irf8^−/−^* zebrafish. Furthermore, treatment with the CSF-1R inhibitor BLZ945 resulted in decreased U87 cell growth as previously observed in a mouse proneural GBM model.^26^

In summary, these experiments show that zebrafish microglia develop a protumoral activity and promote the growth of human glioblastoma cells. This highlights the suitability of the larval zebrafish model to investigate the role of microglia during brain tumor growth.

## Conclusion

Finally, the fact that microglia respond differently depending on the glioma cell type could be very important when developing novel immunotherapeutics to target gliomas. Immunotherapeutics that specifically target microglia interactions may work on certain subtypes of gliomas, but the same desirable effect may not be achieved on others. The zebrafish xenograft assay presented in this study will become a powerful tool in cancer research to understand microglia–glioma interactions in detail *in vivo*. Due to advances in vertebrate animal screening technologies, zebrafish larvae can be screened and imaged automatically in 96-well plate format.^63^ Thus, based on our xenograft live imaging model, drug screening can be performed on a medium to high throughput level to identify drugs that impair the protumoral functions of microglia within gliomas.

## Supplementary Material

Supplemental data

Supplemental data

Supplemental data

Supplemental data

Supplemental data

## References

[1] Holland EC Glioblastoma multiforme: the terminator. Proc Natl Acad Sci USA 2000;97:6242–6244 10841526 10.1073/pnas.97.12.6242 PMC33993

[2] Ostrom QT, Gittleman H, Liao P, Rouse C, Chen Y, Dowling J, *et al.* CBTRUS Statistical Report: primary brain and central nervous system tumors diagnosed in the United States in 2007–2011. Neuro Oncol 2014;16(suppl 4):iv1–iv63 25304271 10.1093/neuonc/nou223 PMC4193675

[3] Agnihotri S, Burrell KE, Wolf A, Jalali S, Hawkins C, Rutka JT, *et al.* Glioblastoma, a brief review of history, molecular genetics, animal models and novel therapeutic strategies. Arch Immunol Ther Exp 2012;61:25–41 10.1007/s00005-012-0203-0 23224339

[4] Popova SN, Bergqvist M, Dimberg A, Edqvist P-H, Ekman S, Hesselager G, *et al.* Subtyping of gliomas of various WHO grades by the application of immunohistochemistry. Histopathology 2013;64:365–379 24410805 10.1111/his.12252 PMC4670475

[5] Scherer HJ A critical review: the pathology of cerebral gliomas. J Neurol Psychiatry 1940;3:147–177 21610973 10.1136/jnnp.3.2.147 PMC1088179

[6] Badie B, Schartner J Role of microglia in glioma biology. Microsc Res Tech 2001;54:106–113 11455617 10.1002/jemt.1125

[7] Graeber MB, Scheithauer BW, Kreutzberg GW Microglia in brain tumors. Glia 2002;40:252–259 12379912 10.1002/glia.10147

[8] Platten M, Kretz A, Naumann U, Aulwurm S, Egashira K, Isenmann S, *et al.* Monocyte chemoattractant protein-1 increases microglial infiltration and aggressiveness of gliomas. Ann Neurol 2003;54:388–392 12953273 10.1002/ana.10679

[9] Coniglio SJ, Segall JE UNCORRECTED PROOF. Matrix Biol 2013;32:372–380 23933178 10.1016/j.matbio.2013.07.008

[10] Carvalho da Fonseca AC, Badie B Microglia and macrophages in malignant gliomas: recent discoveries and implications for promising therapies. Clin Dev Immunol 2013;2013:1–5 10.1155/2013/264124 PMC3707269 23864876

[11] Wu A, Wei J, Kong LY, Wang Y, Priebe W, Qiao W, *et al.* Glioma cancer stem cells induce immunosuppressive macrophages/microglia. Neuro Oncol 2010;12:1113–1125 20667896 10.1093/neuonc/noq082 PMC3098021

[12] Wang H, Zhang L, Zhang IY, Chen X, Carvalho da Fonseca AC, Wu S, *et al.* S100B promotes glioma growth through chemoattraction of myeloid-derived macrophages. Clin Cancer Res 2013;19:3764–3775 23719262 10.1158/1078-0432.CCR-12-3725 PMC3725731

[13] Ellert-Miklaszewska A, Dabrowski M, Lipko M, Sliwa M, Maleszewska M, Kaminska B Molecular definition of the pro-tumorigenic phenotype of glioma-activated microglia. Glia 2013;61:1178–1190 23650109 10.1002/glia.22510

[14] Zhang J, Sarkar S, Cua R, Zhou Y, Hader W, Yong VW A dialog between glioma and microglia that promotes tumor invasiveness through the CCL2/CCR2/interleukin-6 axis. Carcinogenesis 2012;33:312–319 22159219 10.1093/carcin/bgr289

[15] Markovic DS, Glass R, Synowitz M, Rooijen NV, Kettenmann H Microglia stimulate the invasiveness of glioma cells by increasing the activity of metalloprotease-2. J Neuropathol Exp Neurol 2005;64:754–762 16141784 10.1097/01.jnen.0000178445.33972.a9

[16] Li W, Graeber MB The molecular profile of microglia under the influence of glioma. Neuro Oncol 2012;14:958–978 22573310 10.1093/neuonc/nos116 PMC3408253

[17] Yang I, Han SJ, Kaur G, Crane C, Parsa AT The role of microglia in central nervous system immunity and glioma immunology. J Clin Neurosci 2010;17:6–10 19926287 10.1016/j.jocn.2009.05.006 PMC3786731

[18] Kettenmann H, Hanisch UK, Noda M, Verkhratsky A Physiology of microglia. Physiol Rev 2011;91:461–553 21527731 10.1152/physrev.00011.2010

[19] Casano AM, Peri F Microglia: multitasking specialists of the brain. Dev Cell 2015;32:469–477 25710533 10.1016/j.devcel.2015.01.018

[20] Davalos D, Grutzendler J, Yang G, Kim JV, Zuo Y, Jung S, *et al.* ATP mediates rapid microglial response to local brain injury in vivo. Nat Neurosci 2005;8:752–758 15895084 10.1038/nn1472

[21] Haynes SE, Hollopeter G, Yang G, Kurpius D, Dailey ME, Gan W-B, *et al.* The P2Y12 receptor regulates microglial activation by extracellular nucleotides. Nat Neurosci 2006;9:1512–1519 17115040 10.1038/nn1805

[22] Neumann H, Kotter MR, Franklin RJM Debris clearance by microglia: an essential link between degeneration and regeneration. Brain 2009;132(Pt 2):288–295 18567623 10.1093/brain/awn109 PMC2640215

[23] Sieger D, Moritz C, Ziegenhals T, Prykhozhij S, Peri F Long-range Ca2+ waves transmit brain-damage signals to microglia. Dev Cell 2012;22:1138–1148 22632801 10.1016/j.devcel.2012.04.012

[24] Zhai H, Heppner FL, Tsirka SE Microglia/macrophages promote glioma progression. Glia 2010;59:472–485 21264953 10.1002/glia.21117 PMC3080032

[25] Komohara Y, Ohnishi K, Kuratsu J, Takeya M Possible involvement of the M2 anti-inflammatory macrophage phenotype in growth of human gliomas. J Pathol 2008;216:15–24 18553315 10.1002/path.2370

[26] Pyonteck SM, Akkari L, Schuhmacher AJ, Bowman RL, Sevenich L, Quail DF, *et al.* CSF-1R inhibition alters macrophage polarization and blocks glioma progression. Nat Med 2013;19:1264–1272 24056773 10.1038/nm.3337 PMC3840724

[27] Hambardzumyan D, Gutmann DH, Kettenmann H The role of microglia and macrophages in glioma maintenance and progression. Nat Neurosci 2015;19:20–27 10.1038/nn.4185 PMC4876023 26713745

[28] Langenau DM, Feng H, Berghmans S, Kanki JP, Kutok JL, Look AT Cre/lox-regulated transgenic zebrafish model with conditional myc-induced T cell acute lymphoblastic leukemia. Proc Natl Acad Sci USA 2005;102:6068–6073 15827121 10.1073/pnas.0408708102 PMC1087915

[29] Langenau DM Myc-induced T cell leukemia in transgenic zebrafish. Science 2003;299:887–890 12574629 10.1126/science.1080280

[30] Sabaawy HE, Azuma M, Embree LJ, Tsai H-J, Starost MF, Hickstein DD TEL-AML1 transgenic zebrafish model of precursor B cell acute lymphoblastic leukemia. Proc Natl Acad Sci USA 2006;103:15166–15171 17015828 10.1073/pnas.0603349103 PMC1622794

[31] Santoriello C, Deflorian G, Pezzimenti F, Kawakami K, Lanfrancone L, d'Adda di Fagagna F, *et al.* Expression of H-RASV12 in a zebrafish model of Costello syndrome causes cellular senescence in adult proliferating cells. Dis Model Mech 2009;2:56–67 19132118 10.1242/dmm.001016 PMC2615164

[32] Santoriello C, Anelli V, Alghisi E, Mione M Highly penetrant melanoma in a zebrafish model is independent of ErbB3b signaling. Pigment Cell Melanoma Res 2012;25:287–289 22248380 10.1111/j.1755-148X.2012.00973.x

[33] Zeng Z, Richardson J, Verduzco D, Mitchell DL, Patton EE Zebrafish have a competent p53-dependent nucleotide excision repair pathway to resolve ultraviolet B-induced DNA damage in the skin. Zebrafish 2009;6:405–415 20047468 10.1089/zeb.2009.0611 PMC2804931

[34] Patton EE, Widlund HR, Kutok JL, Kopani KR, Amatruda JF, Murphey RD, *et al.* BRAF mutations are sufficient to promote nevi formation and cooperate with p53 in the genesis of melanoma. Curr Biol 2005;15:249–254 15694309 10.1016/j.cub.2005.01.031

[35] Jung IH, Leem GL, Jung DE, Kim MH, Kim EY, Kim SH, *et al.* Glioma is formed by active Akt1 alone and promoted by active Rac1 in transgenic zebrafish. Neuro Oncol 2013;15:290–304 23325864 10.1093/neuonc/nos387 PMC3578497

[36] Shin J, Padmanabhan A, de Groh ED, Lee JS, Haidar S, Dahlberg S, *et al.* Zebrafish neurofibromatosis type 1 genes have redundant functions in tumorigenesis and embryonic development. Dis Model Mech 2012;5:881–894 22773753 10.1242/dmm.009779 PMC3484870

[37] Lal S, La Du J, Tanguay RL, Greenwood JA Calpain 2 is required for the invasion of glioblastoma cells in the zebrafish brain microenvironment. J Neurosci Res 2011;90:769–781 22183788 10.1002/jnr.22794 PMC3274595

[38] Welker AM, Jaros BD, Puduvalli VK, Imitola J, Kaur B, Beattie CE Standardized orthotopic xenografts in zebrafish reveal glioma cell line specific characteristics and tumor cell heterogeneity. Dis Model Mech 2015;9:1063–1065 10.1242/dmm.027235 PMC5047695 27604693

[39] Renshaw SA, Trede NS A model 450 million years in the making: zebrafish and vertebrate immunity. Dis Model Mech 2011;5:38–47 10.1242/dmm.007138 PMC3255542 22228790

[40] Paik EJ, Zon LI Hematopoietic development in the zebrafish. Int J Dev Biol 2010;54:1127–1137 20711990 10.1387/ijdb.093042ep

[41] Palmer GM, Fontanella AN, Shan S, Hanna G, Zhang G, Fraser CL, *et al.* In vivo optical molecular imaging and analysis in mice using dorsal window chamber models appliedto hypoxia, vasculature and fluorescent reporters. Nat Protoc 2011;6:1355–1366 21886101 10.1038/nprot.2011.349 PMC3500601

[42] Maes W, Van Gool SW Experimental immunotherapy for malignant glioma: lessons from two decades of research in the GL261 model. Cancer Immunol Immunother 2010;60:153–160 21120655 10.1007/s00262-010-0946-6 PMC11028904

[43] Resende FFB, Bai X, Del Bel EA, Kirchhoff F, Scheller A, Titze-de-Almeida R Evaluation of TgH(CX3CR1-EGFP) mice implanted with mCherry-GL261 cells as an in vivo model for morphometrical analysis of glioma-microglia interaction. BMC Cancer 2016;16:72 26856327 10.1186/s12885-016-2118-3 PMC4746826

[44] Bayerl SH, Niesner R, Cseresnyes Z, Radbruch H, Pohlan J, Brandenburg S, *et al.* Time lapse in vivo microscopy reveals distinct dynamics of microglia-tumor environment interactions—a new role for the tumor perivascular space as highway for trafficking microglia. Glia 2016;64:1210–1226 27143298 10.1002/glia.22994

[45] Ricard C, Tchoghandjian A, Luche H, Grenot P, Figarella-Branger D, Rougon G, *et al.* Phenotypic dynamics of microglialand monocyte-derived cells inglioblastoma-bearing mice. Sci Rep 2016;6:1–15 10.1038/srep26381 PMC4872227 27193333

[46] Ellett F, Pase L, Hayman JW, Andrianopoulos A, Lieschke GJ mpeg1 promoter transgenes direct macrophage-lineage expression in zebrafish. Blood 2011;117:e49–e56 21084707 10.1182/blood-2010-10-314120 PMC3056479

[47] Shiau CE, Kaufman Z, Meireles AM, Talbot WS Differential requirement for irf8 in formation of embryonic and adult macrophages in zebrafish. PLoS One 2015;10:e0117513 25615614 10.1371/journal.pone.0117513 PMC4304715

[48] Peri F, Nüsslein-Volhard C Live Imaging of neuronal degradation by microglia reveals a role for v0-ATPase a1 in phagosomal fusion in vivo. Cell 2008;133:916–927 18510934 10.1016/j.cell.2008.04.037

[49] Svahn AJ, Graeber MB, Ellett F, Lieschke GJ, Rinkwitz S, Bennett MR, *et al.* Development of ramified microglia from early macrophages in the zebrafish optic tectum. Dev Neurobiol 2012;73:60–71 22648905 10.1002/dneu.22039

[50] Li Y, Du X-F, Liu C-S, Wen Z-L, Du J-L Reciprocal regulation between resting microglial dynamics and neuronal activity in vivo. Dev Cell 2012;23:1189–1202 23201120 10.1016/j.devcel.2012.10.027

[51] Radaelli E, Ceruti R, Patton V, Russo M, Degrassi A, Croci V, *et al.* Immunohistopathological and neuroimaging characterization of murine orthotopic xenograft models of glioblastoma multiforme recapitulating the most salient features of human disease. Histol Histopathol 2009;24:879–891 19475534 10.14670/HH-24.879

[52] Jacobs VL, Valdes PA, Hickey WF, De Leo JA Current review of in vivoGBM rodent models: emphasis on the CNS-1 tumour model. ASN Neuro 2011;3:171–181 10.1042/AN20110014 PMC3153964 21740400

[53] Candolfi M, Curtin JF, Nichols WS, Muhammad AG, King GD, Pluhar GE, *et al.* Intracranial glioblastoma models in preclinical neuro-oncology: neuropathological characterization and tumor progression. J Neurooncol 2007;85:133–148 17874037 10.1007/s11060-007-9400-9 PMC2384236

[54] Geiger GA, Fu W, Kao GD Temozolomide-mediated radiosensitization of human glioma cells in a zebrafish embryonic system. Cancer Res 2008;68:3396–3404 18451167 10.1158/0008-5472.CAN-07-6396 PMC3616326

[55] Lally BE, Geiger GA, Kridel S, Arcury-Quandt AE, Robbins ME, Kock ND, *et al.* Identification and biological evaluation of a novel and potent small molecule radiation sensitizer via an unbiased screen of a chemical library. Cancer Res 2007;67:8791–8799 17875720 10.1158/0008-5472.CAN-07-0477 PMC3610568

[56] Yang X-J, Cui W, Gu A, Xu C, Yu S-C, Li T-T, *et al.* A novel zebrafish xenotransplantation model for study of glioma stem cell invasion. PLoS One 2013;8:e61801 23613942 10.1371/journal.pone.0061801 PMC3628966

[57] Kitambi SS, Toledo EM, Usoskin D, Wee S, Harisankar A, Svensson R, *et al.* Vulnerability of glioblastoma cellsto catastrophic vacuolization and death induced by a small molecule. Cell 2014;157:313–328 24656405 10.1016/j.cell.2014.02.021

[58] Hruz T, Laule O, Szabo G, Wessendorp F, Bleuler S, Oertle L, *et al.* Genevestigator V3: a reference expression database for the meta-analysis of transcriptomes. Adv Bioinformatics 2008;2008:1–5 10.1155/2008/420747 PMC2777001 19956698

[59] Chang Y Lymphotoxin β receptor induces interleukin 8 gene expression via NF-κB and AP-1 activation. Exp Cell Res 2002;278:166–174 12169272 10.1006/excr.2002.5573

[60] Suwara MI, Green NJ, Borthwick LA, Mann J, Mayer-Barber KD, Barron L, *et al.* Released from damaged epithelial cells issufficient and essential to trigger inflammatory responses in human lung fibroblasts. Nature 2013;7:684–693 10.1038/mi.2013.87 PMC3931585 24172847

[61] Verhaak RGW, Hoadley KA, Purdom E, Wang V, Qi Y, Wilkerson MD, *et al.* Integrated genomic analysis identifies clinically relevant subtypes of glioblastoma characterized by abnormalities in PDGFRA, IDH1, EGFR, and NF1. Cancer Cell 2010;17:98–110 20129251 10.1016/j.ccr.2009.12.020 PMC2818769

[62] Doucette T, Rao G, Rao A, Shen L, Aldape K, Wei J, *et al.* Immune heterogeneity of glioblastoma subtypes: extrapolation from the cancer genome atlas. Cancer Immunol Res 2013;1:112–122 24409449 10.1158/2326-6066.CIR-13-0028 PMC3881271

[63] Pardo-Martin C, Chang T-Y, Koo BK, Gilleland CL, Wasserman SC, Yanik MF high-throughput. Nat Methods 2010;7:634–636 20639868 10.1038/nmeth.1481 PMC2941625

