# Supplementary material for: A Zebrafish Live Imaging Model Reveals Differential Responses of Microglia Toward Glioblastoma Cells In Vivo
Source: Zebrafish. 2016 Dec 1;13(6):523–34. doi: 10.1089/zeb.2016.1339 (PMC5124743; doi:10.1089/zeb.2016.1339)
Supplement: Supplemental data [file Supp_Fig1.pdf]

Supplementary Data

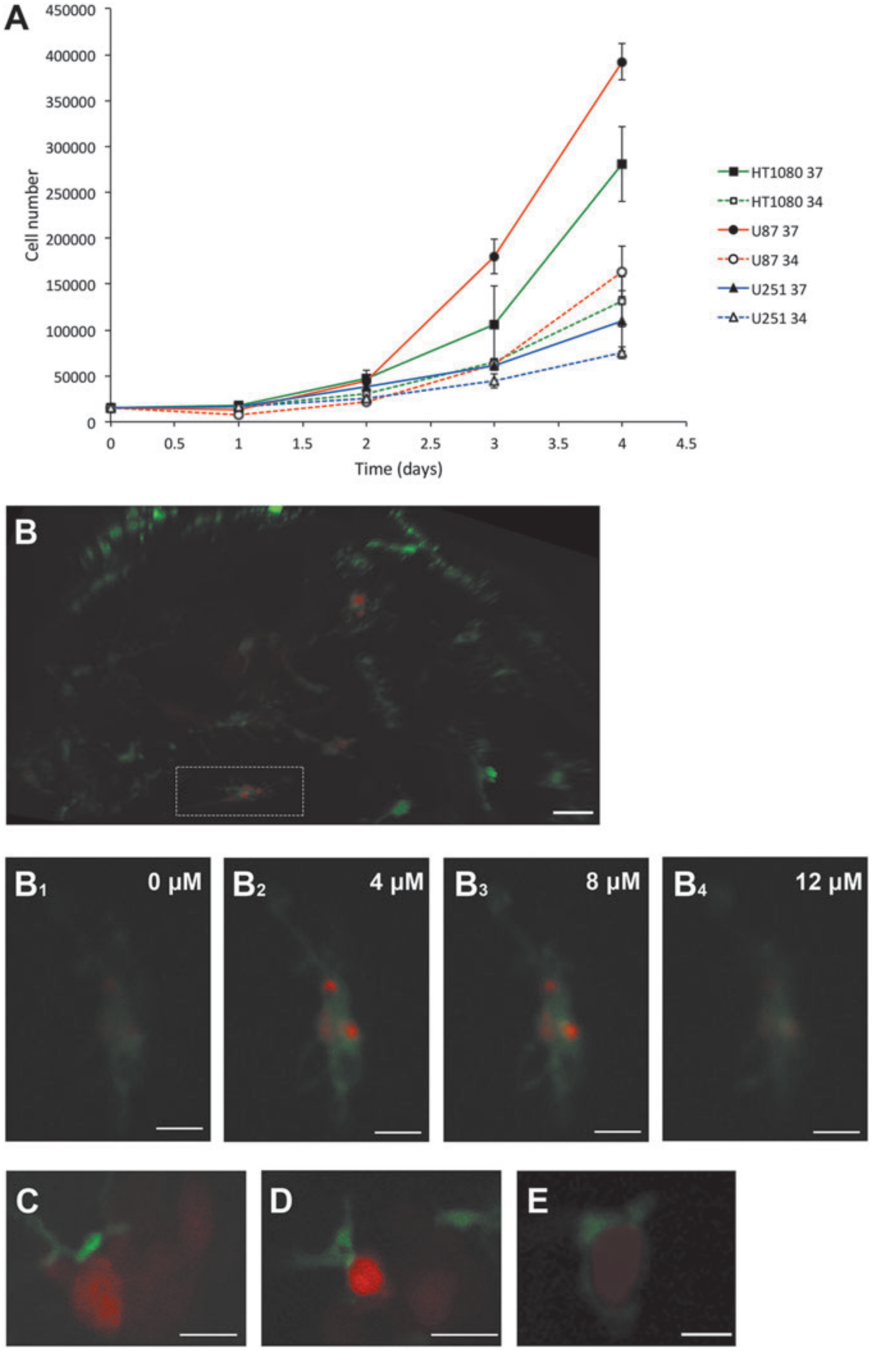

SUPPLEMENTARY FIG. S1. *In vitro* growth of U251, U87, and HT1080 cells and microglial interactions upon cell transplantation. **(A)** The growth rates of HT1080 mCherry, U87 mCherry, and U251 mCherry cells at 34°C compared to 37°C over a 4-day period. The experiment was repeated in triplicate, and the average cell numbers, from the three experiments, are plotted. **(B)** Representative confocal image of the optic tectum of mpeg1:EGFP zebrafish at 3 dpt with HT1080mCherry fibrosarcoma cells. Image represents a maximum intensity projection of a confocal stack. *White rectangle* encompasses the microglial cell that is shown in **(B_1–B_4)**. **(B_1–B_4)** Single confocal z sections showing HT1080 cell fragments (*red*) engulfed by microglia (*green*). Confocal z sections are in a distance of 4 μm from each other. Images were captured using an Andor spinning disk confocal microscope with a 20×/NA 0.75 objective. Scale bar for **(B)**: 30 μm, scale bar for **(B_1–B_4)**: 8 μm. **(C)** Single confocal z section showing a microglial cell (*green*) directly interacting with a U251 cell (*red*). Scale bar 15 μm. **(D)** Single confocal z section showing a microglial cell (*green*) directly interacting with a U87 cell (*red*). Scale bar 20 μm. **(E)** Single confocal z section showing a microglial cell (*green*) directly interacting with a HT1080 cell (*red*). Scale bar 10 μm. **(C–E)** Images were captured using an Andor spinning disk confocal microscope with a 20×/NA 0.75 objective.
