# Supplementary material for: A Zebrafish Live Imaging Model Reveals Differential Responses of Microglia Toward Glioblastoma Cells In Vivo
Source: Zebrafish. 2016 Dec 1;13(6):523–34. doi: 10.1089/zeb.2016.1339 (PMC5124743; doi:10.1089/zeb.2016.1339)
Supplement: Supplemental data [file Supp_Fig2.pdf]

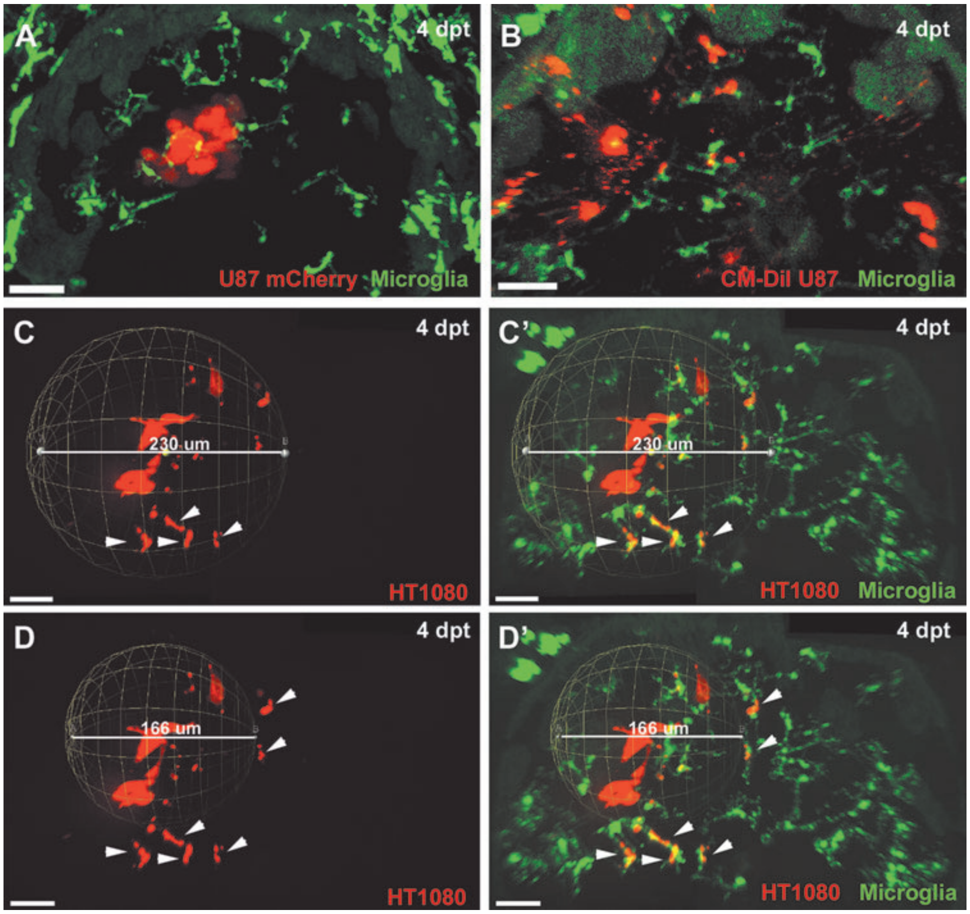

SUPPLEMENTARY FIG. S2. Prerequirements to measure invasiveness precisely: fluorescent protein expression in transplanted cells and microglia labelling. CM-DiI Dye labelled cells showed false positive rates of invasiveness due to detection of small fluorescent particles that might be either pure dye aggregates or vesicles released by tumor cells. **(A)** Confocal image of the optic tectum of mpeg1:EGFP zebrafish transplanted with U87 glioblastoma cells stably expressing the mCherry fluorescent protein (4 dpt). **(B)** Confocal image of the optic tectum of mpeg1:EGFP zebrafish transplanted with CM-DiI Dye labeled U87 glioblastoma cells (4 dpt). Microglia need to be taken into account to exclude transplanted cells that are within phagosomes of microglia and, thus, not viable and not truly invasive. **(C, D)** Confocal images of the optic tectum of mpeg1:EGFP zebrafish transplanted with HT1080mCherry cells (4 dpt). **(C′, D′)** Confocal images of the optic tectum of mpeg1:EGFP zebrafish transplanted with HT1080mCherry cells (same images as **C, D**) showing the mpeg1:EGFP channel in addition. **(C, C′)** Sphere measurement for invasiveness, including all HT1080mCHerry cells. **(D, D′)** Sphere measurement for invasiveness excluding HT1080mCherry cells within microglia. HT1080 cells within microglia are marked with a *white arrowhead*. Scale bars for all images: 30 μm. All images represent maximum intensity projections of confocal stacks. Images were captured using an Andor spinning disk confocal microscope with a 20×/NA 0.75 objective.
