# Supplementary material for: A Zebrafish Live Imaging Model Reveals Differential Responses of Microglia Toward Glioblastoma Cells In Vivo
Source: Zebrafish. 2016 Dec 1;13(6):523–34. doi: 10.1089/zeb.2016.1339 (PMC5124743; doi:10.1089/zeb.2016.1339)
Supplement: Supplemental data [file Supp_Movie3.zip › Supp_Movie3.pdf]

SUPPLEMENTARY MOVIE S3. Time-lapse movie showing 17 h of representative microglial behavior (*green*) after HT1080 cell transplantation (*red*). Images were captured every 3 min using an Andor spinning disk confocal microscope with a 20×/NA 0.75 objective.
